# Mechanical Thrombectomy in Ischemic Stroke with a Large Infarct Core: A Meta-Analysis of Randomized Controlled Trials

**DOI:** 10.3390/jcm13154280

**Published:** 2024-07-23

**Authors:** Michele Romoli, Lucia Princiotta Cariddi, Marco Longoni, Gianluca Stufano, Sebastiano Giacomozzi, Luca Pompei, Francesco Diana, Lucio D’Anna, Simona Sacco, Simone Vidale

**Affiliations:** 1 Department of Neurosciences, AUSL Romagna, Bufalini Hospital, 47521 Cesena, Italy; 2 Department of Neurology, ASST Sette Laghi, 21100 Varese, Italysimone.vidale@asst-settelaghi.it (S.V.); 3 Neuroradiologia Intervencionista, Hospital Universitari Vall d’Hebron, 08035 Barcelona, Spain; 4 Department of Stroke and Neuroscience, Charing Cross Hospital, Imperial College London NHS Healthcare Trust, London W6 8RF, UK; 5 Department of Brain Sciences, Imperial College London, London W6 8RF, UK; 6 Department of Biotechnological and Applied Clinical Sciences, University of L’Aquila, 67100 L’Aquila, Italy

**Keywords:** large ischemic stroke, mechanical thrombectomy, meta-analysis, RCTs

## Abstract

**Background/Objectives:** Endovascular treatment (EVT) is recommended for acute ischemic stroke due to large-vessel occlusion (LVO) and an Alberta Stroke Program Early CT Score (ASPECTS) ≥ 6. Randomized controlled trials (RCTs) have recently become available on EVT effects in people with LVO-related large core stroke (ASPECTS 0–5). Here, we provide an updated meta-analysis of the EVT effect on functional neurological status in people with large-core stroke. **Methods:** The study followed the PRISMA guidelines. PubMed, EMBASE and Cochrane Central were searched for RCTs comparing EVT vs. best medical treatment (BMT) in large-core LVO stroke. The primary outcome was functional independence at 90 days (modified Rankin Scale; mRS 0–2). The secondary outcomes were symptomatic intracranial hemorrhage (sICH), good functional outcome (mRS 0–3) and excellent functional outcome (mRS 0–1). EVT vs. BMT was compared through random effect meta-analysis. Heterogeneity was assessed with the I^2^ and Q test and risk of bias reported according to the RoB2 tool. **Results:** Six RCTs were included (n = 1656 patients). All studies had a moderate risk of bias, with blinding bias due to the nature of the intervention, potential allocation bias and incomplete outcome reporting. Functional independence was significantly more frequent in the EVT vs. BMT group (OR = 2.47, 95% CI = 1.52–4.03, *p* < 0.001). sICH rates (OR = 1.77, 95% CI = 1.01–3.11, *p* = 0.04) and good functional outcome (OR = 2.20; 95% CI = 1.72–2.81, *p* < 0.001) were more frequent in the EVT vs. BMT group, while the rates of mRS 0–1 did not differ. **Conclusions:** In patients with large-core stroke and LVO, EVT plus BMT as compared to BMT alone carries a significant increase in independent ambulation and good functional outcome at 3 months despite the marginal increase in sICH.

## 1. Introduction

Mechanical thrombectomy (EVT) with or without intravenous thrombolysis (IVT) is effective in improving the functional outcome in ischemic stroke patients with large vessel occlusion (LVO) [1,2]. According to the current guidelines, neuroradiological features are among the critical factors to define eligibility to revascularization treatments [1,2]. An Alberta Stroke Program Computed Tomography Score (ASPECTS) > 5 or the presence of a significant mismatch area between the infarct core and perfusion deficit are needed to determine the eligibility for EVT [1,2]. Such criteria derived from pivotal randomized clinical trials (RCTs) and implied the exclusion of large-core LVO-related stroke cases from reperfusion treatment.

Recently, randomized controlled trials (RCT) have also suggested the benefit of EVT in stroke with a large established core infarct [3,4,5,6,7]. Such results conflict with those previously reported from mixed observational and clinical trials [8] and may therefore require an attempt at synthesis to derive treatment effect estimates. Indeed, previous meta-analysis [9] did not include all relevant RCTs [3,4,5,6,7], limiting the interpretation of the treatment estimates provided but also allowing refinement through additional high-quality data. 

We performed a systematic review and meta-analysis of RCTs investigating the efficacy and safety of EVT of large-core infarct stroke.

## 2. Methods

### 2.1. Search Strategy 

**Figure 1:**
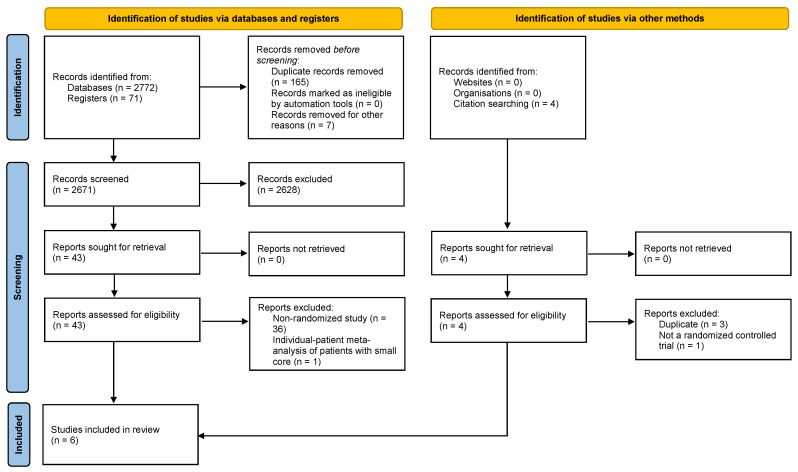
PRISMA flowchart for the study selection.

The methods and guidelines of this study-level meta-analysis followed the PRISMA [10] guidelines, and the study protocol was deposited with OSF (DOI: 10.17605/OSF.IO/CPW97). Two reviewers systematically searched PubMed, EMBASE and the Cochrane Central register of Controlled Trials for studies investigating the efficacy and safety of EVT in large-core ischemic strokes and published between January 1990 and February 2024. The search strategy included the combination of terms for stroke, thrombectomy and large core (Supplementary Materials). The reference lists and cited articles were also reviewed to increase the identification of relevant studies. Two reviewers screened and revised the result list and selected studies for full evaluation (Figure 1), with disagreements resolved by consensus. 

### 2.2. Inclusion Criteria and Data Extraction

In this pooled analysis, we included only RCTs comparing the clinical efficacy and safety of EVT or combined treatment with intravenous thrombolysis among adult (≥18) patients with acute ischemic stroke due to LVO and with established large-core infarct (ASPECTS < 6). We limited the studies to the English language. The interventional group comprised patients treated with EVT with or without IVT, while the control group was represented by patients treated only with best medical treatment (BMT). Two reviewers independently extracted data concerning the baseline features, setting, neuroradiological features and outcome characteristics of each included study. We reported the lack of data on the outcome when appropriate.

### 2.3. Outcomes

The primary endpoint was functional independence at 90 days from stroke onset, defined as the modified Rankin Scale (mRS) 0-2. The secondary endpoints were (i) symptomatic intracranial hemorrhage (sICH), defined according to trial-specific adjudication criteria, (ii) good functional outcome (mRS 0-3) and (iii) excellent functional outcome (mRS 0-1). Ordinal shift analysis for the mRS scores was also reported. The risk of bias was assessed and reported according to the recommendations of the Cochrane Handbook for Systematic Reviews of Intervention. 

### 2.4. Statistical Analysis

We performed a statistical analysis by pooling the data in the intervention group and the control group. Heterogeneity was evaluated with Cochrane’s Q test and I^2^, with fixed and random effects models applied accordingly. We pooled the data from the intervention group and control group, reporting the results through odds ratios (ORs) and 95% confidence intervals (CIs) for all outcomes and using Forest plots for graphical representation. A sensitivity analysis was planned for studies not using CT perfusion imaging. Data analysis was performed using RevMan 5.3 (The Cochrane Collaboration 2012

## 3. Results

We identified and screened 2671 records from a systematic search, finally including six RCTs with a total of 1656 patients in the analysis (PRISMA flowchart, Figure 1) [3,4,5,6,7,11]. 

Table 1 summarizes the characteristics of each study and the respective risk of bias. All included studies lacked the blinding of patients and investigators due to the intervention itself and had minor deviations from the intended intervention. We detected a very low risk of bias for the outcome assessment and reporting the results for all the studies. A risk of bias also emerged in two RCTs [3,4] in relation to the missing data on patients lost to follow-up (Table 1). No significant differences emerged for cardiovascular risk factors distribution across the EVT vs. BMT groups (Supplementary Materials Table S1). 

**Figure 2:**
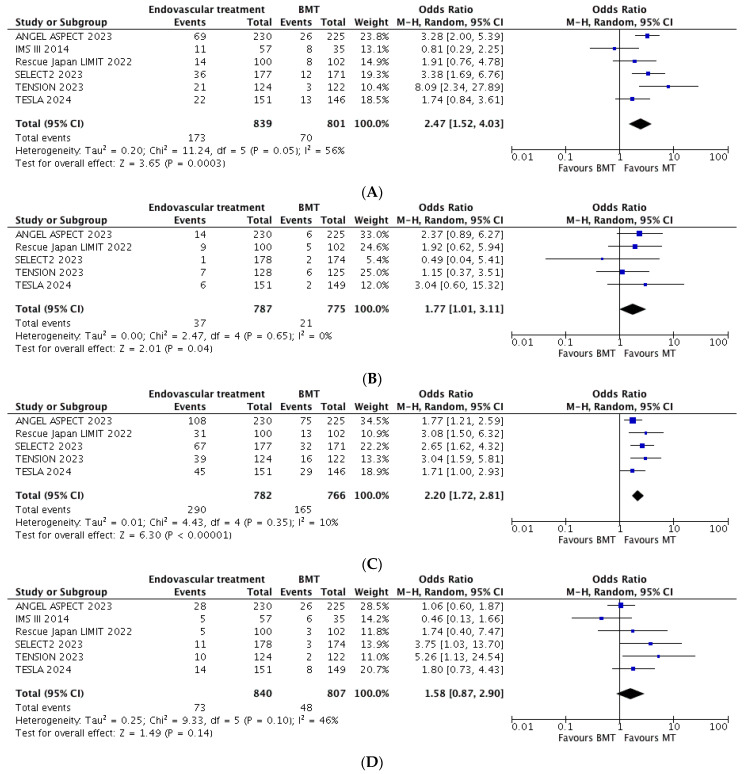
Pooled estimate for functional independence (mRS 0–2, (**A**)), symptomatic intracranial hemorrhage (sICH, (**B**)), good functional outcome (mRS 0–3, (**C**)) and excellent outcome (mRS 0–1, (**D**)).

Pooling the results from all six studies included, functional independence was achieved in 20.6% of cases in the EVT group vs. 8.7% of cases in the BMT group (OR = 2.47, 95% CI = 1.52–4.03, *p* < 0.001) (Figure 2A). 

Pooling the data from five studies, the sICH was marginally more frequent in the EVT vs. BMT group (4.7% vs. 2.7%; OR = 1.77, 95% CI = 1.01–3.11, *p* = 0.04) (Figure 2B). A good functional outcome (mRS 0-3) was more frequent among the people receiving EVT compared to those receiving BMT (37% vs. 21.5%; OR = 2.20, 95% CI = 1.72–2.81, *p* < 0.001) (Figure 2C). Sensitivity confirmed a significant benefit from EVT in terms of good functional outcome (mRS 0-3) over BMT and, also, among studies with a baseline selection through non-contrast brain CT only (OR = 2.39, 95% CI = 1.60–3.58) (Supplementary Materials Figure S1).

Pooling the data from all six studies included in the analysis, the excellent functional outcome (mRS 0-1) was similar across the groups (8.7% for the EVT vs. 6% for the BMT group; OR = 1.58, 95% CI = 0.87–2.90, *p* = 0.14) (Figure 2D). 

**Figure 3:**
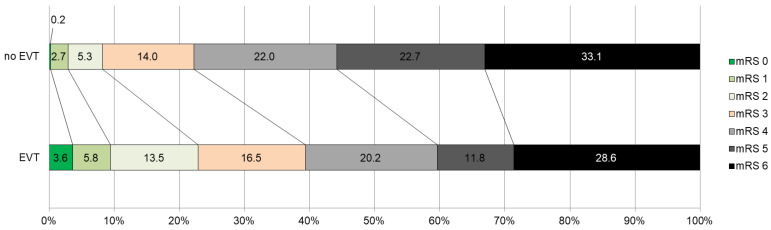
Distribution of the mRS scores in the EVT and no EVT groups.

Across all six included studies, the distribution of mRS scores at 90 days of follow-up showed a significant benefit of EVT compared to BMT (generalized OR = 1.62, 95% CI = 1.38–1.90, *p* = 0.03) (Figure 3).

## 4. Discussion

The results of this meta-analysis of RCT data highlight that, in patients with large core infarct and LVO, EVT plus BMT as compared to BMT alone carries a significant increase in the chances of achieving an independent ambulation and good functional outcome at 3 months. Pooling the data from six RCTs, EVT was associated with a higher chance of independent ambulation at 3 months (OR = 2.47, 95% CI = 1.52–4.03) and of achieving a good functional outcome (OR = 2.20; 95% CI = 1.72–2.81), with a marginally higher risk of sICH after the procedure. The limited heterogeneity across the estimates highlights the treatment effect across studies, despite slight differences in treatment window, baseline NIHSS and ASPECTS entry criteria (Table 1). Even when adding the most recent RCT available (LASTE) [12], left out from the main analysis being published after the search end date, the global estimate of EVT effects and heterogeneity would still be confirmed (Supplementary Materials Table S2). 

Our results add and put into context previous studies and meta-analyses that were limited by the availability of RCTs [13,14] and/or by the quality of available observational studies [9,15,16]. The observational data included in the meta-analysis were derived from studies with consistent variability in the neuroradiological criteria for inclusion, ranging from ASPECTS 6, with a clear indication to treatment, to ASPECTS 0, therefore unlikely to have any benefit from intervention [9,15,16]. RCTs seem to have similar inclusion criteria, and although differing in treatment selection modalities—SELECT2 and ANGEL-ASPECT also used CT perfusion thresholds—only marginal heterogeneity emerges. To this extent, it is important to notice that our estimates for treatment effects slightly differed from those provided in previous attempts at synthesis, mainly in relation to the number and types of studies included. A previous meta-analysis of RCTs [9] including only three trials (RESCUE-JAPAN, SELECT2 and ANGEL-ASPECT) [4,5,6], although estimating a positive treatment effect for EVT regarding mRS 0-3, did not provide data on an excellent outcome (mRS 0-1) and had a nonsignificant increase in sICH rates in the EVT arm [9]. As the overall sample size nearly doubled in this meta-analysis compared to the latter, there now seems to be robust data supporting a positive treatment effect for mRS 0-2 and mRS 0-3, despite a significant increase in the sICH rates and no effect on the excellent outcome, with cases of mRS 0-1 being extremely limited in both groups. This highlights a ceiling effect for EVT in large-core stroke intrinsic to the condition undergoing intervention. Such information can be paramount to inform patients and relatives, as well as to guide expectations of the treatment effect. A larger infarction at the baseline necessarily translates into the need to reconsider what a clinically meaningful outcome can be; therefore, it seems necessary to convey the information that EVT can, at best, provide a higher chance of some recovery but hardly bring the patient back to their functional status before the stroke. 

From a logistic point of view, it should be noted that the RCTs included implemented a treatment window of 6–24 h largely based on NCCT only, with potentially no need for advanced imaging to define the eligibility for EVT. To this extent, it is of note that, although very large infarct core patients (ASPECTS < 3) were excluded from RESCUE-JAPAN [6] and underrepresented in the remaining trials, there also seemed to be preliminary evidence of a treatment effect in this critically ill subgroup [17]. Also, recent RCTs [3,4,5,6,7] seem to have higher rates of good functional outcome compared to older trials [11], including people with ASPECTS 0-4, a trend potentially supported by the evolution of time metrics and technical devices. From an implementation perspective, we should also consider that all RCTs were conducted in comprehensive stroke centers with large experience in EVT techniques and relatively fast transit from diagnostics to the interventional suite. Therefore the caseload and experience may indeed have played a role in determining a rate of sICH as low as 4.7% in EVT-treated individuals with a large core [3,4,5,6,7,11]. 

### Limitations

Our meta-analysis provides estimates of the EVT treatment effect using data from RCTs, with some limitations. First, this is a study-level meta-analysis and therefore limited to available data from RCTs. Second, as per the ASPECTS group data not being available for all studies, the treatment effect based on single-point ASPECTS could not be calculated. At the same time, since the entry criteria were slightly different across trials, the treatment effect among homogenous groups of patients will only be calculated through individual patient data analysis. Third, it should be underlined that all trials had the same limitation regarding treatment blinding, an issue that seems hard to limit given the very nature of the condition. Fourth, as we also included IMS-III RCTs [11], our estimates for the benefit from EVT may appear slightly reduced, as such RCTs counted on time metrics and devices dating back to the 2014 era. Finally, as stroke networks may need revision to also catch patients with a large core and late presentation, cost-effectiveness analyses are needed to provide sustainable policies for care. 

## 5. Conclusions

Overall, the results from this meta-analysis highlight that there is sufficient evidence from RCTs to support the treatment of large-core ischemic stroke associated with LVO in patients selected with NCCT, with potential simplification of the stroke imaging pathway in these cases. Guidelines should revise the certainty of the evidence and update their recommendations for EVT according to the new RCTs available.

## Figures and Tables

**Table 1:** Characteristics of the included studies and risk of bias according to the Cochrane RoB2 tool.

Study	Country/Inclusion Period	Study Inclusion Criteria and Procedures								End of Trial	Potantial Bias in					
		Total Sample (Female)	mRS Baseline	Year Range (Median)	NIHSS Score	Timing of Treatment	Large Core Definition	Imaging	Occlusion Site		Randomization Process (D1)	Deviations from Intended Interventions (D2)	Missing Outcome Data (D3)	Measurement of the Outcome (D4)	Selection of the Reported Results (D5)	Overall Bias
ANGEL ASPECT	China 2020–2022	456 (38.7%)	0–1	18–80 (68)	6–30	24 h	ASPECTS 3-5 (or core volume 70–100 mL)	NCCT, CTP	ICA, M1	Stopped for efficacy after interim analysis	+	−	+	+	+	−
IMS III	USA 2006–2013	92 (49%)	0–2	18–83 (67)	≥10 (or 8–9 with CTA evidence of LVO)	3 h	ASPECTS 0–4	NCCT, CTA	ICA, M1	Halted for futility after interim analysis	+	−	+	+	+	−
RESCUE JAPAN	Japan 2018–2021	203 (44.3%)	0–1	>18 (76)	≥6	6 h (24 h if no ischemic changes on FLAIR imaging)	ASPECTS 3-5	NCCT, CTA or MRI	ICA, M1	Completed as planned	+	−	+	+	+	−
SELECT 2	USA, Canada, Europe, Australia 2019–2022	352 (41.1%)	0–1	18–85 (67)	≥6	24 h	ASPECTS 3-5 (or core ≥ 50 mL)	NCCT, CTP	ICA, M1	Stopped for efficacy after interim analysis	+	−	−	+	+	−
TENSION	Europe, Canada 2018–2023	253 (48.6%)	0–2	≥18 (74)	≤26	12 h	ASPECTS 3-5	NCCT, CTA or MRI	ICA, M1	Stopped for efficacy after interim analysis	+	−	+	+	+	−
TESLA	USA 2019–2022	300 (NA)	0–1	18–85 (NA)	≥6	24 h	ASPECTS 2-5	NCCT, CTA	ICA, M1	Completed as planned	+	−	+	−	+	−

Legend. green (+) for low risk of bias, yellow (−) from some risk of bias.

## Data Availability

The data can be shared from the corresponding author upon request.

## Supplementary Materials

The following supporting information can be downloaded at https://www.mdpi.com/article/10.3390/jcm13154280/s1: Search string, Tables S1 and S2 and Figure S1.
