# Supplementary material for: Mechanical Thrombectomy in Ischemic Stroke with a Large Infarct Core: A Meta-Analysis of Randomized Controlled Trials
Source: J Clin Med. 2024 Jul 23;13(15):4280. doi: 10.3390/jcm13154280 (PMC11313619; doi:10.3390/jcm13154280)
Supplement: Supplementary file 1 [file jcm-13-04280-s001.zip › jcm-2970702-supplementary.pdf]

SUPPLEMENTARY MATERIAL

Search string

((cerebrovasc*) OR (stroke*)) AND ((mechanic*) OR (thrombec*) OR (endovasc*) OR (thrombectomy [MeSH])) AND ((large) AND ((core) OR (infarct)))

Table S1: Baseline characteristics between interventional and control group*

	MT	BMT
**Number**	808	794
**Median Age (yrs)**	70	71
**Male**	432	437
**Median NIHSS onset**	18	17
**Hypertension**	548	542
**Diabetes**	190	169
**Dyslipidemia**	81	81
**AF**	243	236
**ASPECTS value**		
**0 - 2**	52	56
**3**	185	195
**4**	137	118
**5**	100	109

* no data available for IMS III trial; no ASPECT Score data available for SELECT2 and TESLA trials.

Figure S1. Sensitivity analysis for functional independence (A) and good functional outcome (B) including only studies that did not rely on perfusion imaging for patient inclusion.

A. mRS 0-2

Study or Subgroup	Endovascular treatment Events	Total	BMT Events	Total	Weight	Odds Ratio M-H, Random, 95% CI
IMS III 2014	11	57	8	35	23.9%	0.81 [0.29, 2.25]
Rescue Japan LIMIT 2022	14	100	8	102	26.0%	1.91 [0.76, 4.78]
TENSION 2023	21	124	3	122	20.2%	8.09 [2.34, 27.89]
TESLA 2024	22	151	13	146	29.9%	1.74 [0.84, 3.61]
**Total (95% CI)**		**432**		**405**	**100.0%**	**2.03 [0.92, 4.45]**
Total events	68		32			

Heterogeneity: Tau² = 0.40; Chi² = 8.14, df = 3 (P = 0.04); I² = 63%
Test for overall effect: Z = 1.76 (P = 0.08)

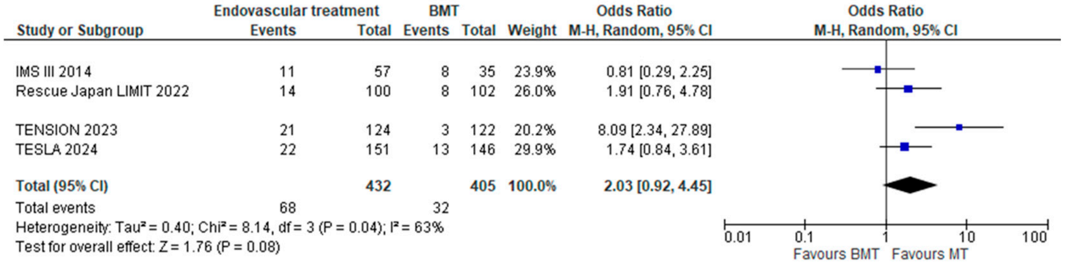

B. mRS 0-3

Study or Subgroup	Endovascular treatment Events	Total	BMT Events	Total	Weight	Odds Ratio M-H, Random, 95% CI
Rescue Japan LIMIT 2022	31	100	13	102	26.4%	3.08 [1.50, 6.32]
TENSION 2023	39	124	16	122	31.4%	3.04 [1.59, 5.81]
TESLA 2024	45	151	29	146	42.3%	1.71 [1.00, 2.93]
**Total (95% CI)**		**375**		**370**	**100.0%**	**2.39 [1.60, 3.58]**
Total events	115		58			

Heterogeneity: Tau² = 0.02; Chi² = 2.48, df = 2 (P = 0.29); I² = 19%
Test for overall effect: Z = 4.25 (P < 0.0001)

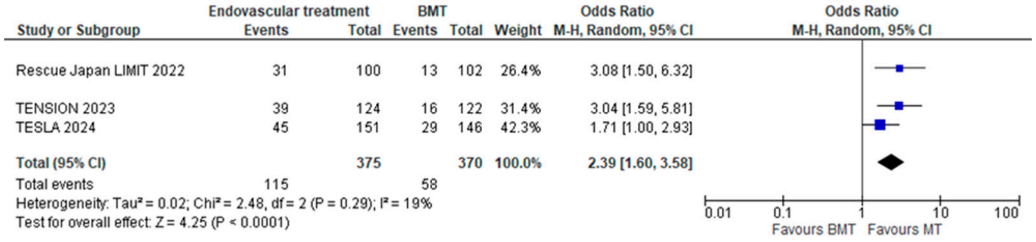

Table S2 Change in estimates including the LASTE trial – published after end-date of systematic search -.

	**without LASTE**		**with LASTE**	
	OR estimate (95%CI)*	I^2	OR estimate (95%CI)*	I^2
**mRS 0-2**	2.47 (1.52-4.03)	56%	2.55 (1.68-3.86)	47%
**sICH**	1.77 (1.01-3.11)	0%	1.61 (0.97-2.69)	0%
**mRS 0-3**	2.20 (1.72-2.81)	10%	2.02 (1.50-2.74)	46%
**mRS 0-1**	1.58 (0.87-2.90)	46%	1.65 (0.96-2.83)	40%
**mRS shift analysis (generalized OR)**	1.62 (1.38-1.90)	33%	1.59 (1.33-1.82)	42%
